# Inhibition of LSD1 promotes the differentiation of human induced pluripotent stem cells into insulin-producing cells

**DOI:** 10.1186/s13287-020-01694-8

**Published:** 2020-05-19

**Authors:** Xiao-Fei Yang, Shu-Yan Zhou, Ce Wang, Wei Huang, Ning Li, Fei He, Fu-Rong Li

**Affiliations:** 1 Translational Medicine Collaborative Innovation Center, The Second Clinical Medical College (Shenzhen People’s Hospital), Ji’nan University, 1017 Dongmen North Road, Shenzhen, 518020 China; 2 Guangdong Engineering Technology Research Center of Stem Cell and Cell therapy, Shenzhen, 518020 China; 3 Shenzhen Cell Therapy Public Service Platform, Shenzhen, 518020 China; 4 Department of Biology, Southern University of Science and Technology, Shenzhen, 518055 China

**Keywords:** LSD1, Human induced pluripotent stem cells, Insulin-producing cells, Differentiation, Diabetes

## Abstract

**Background:**

Human induced pluripotent stem cells (hiPSCs) represent a potentially unlimited source of pancreatic endocrine lineage cells. Although insulin-producing β cells derived from hiPSCs have been successfully induced, much work remains to be done to achieve mature β cells. Lysine-specific demethylase 1 (LSD1) plays an important role in the regulation of hiPSC self-renewal and differentiation. We propose a new strategy to acquire insulin-producing cells (IPCs) from hiPSCs by knocking down LSD1.

**Methods:**

Knockdown of LSD1 in hiPSCs with five shRNA. Assessment of the effects of shRNA on hiPSC proliferation, cell cycle, and apoptosis. Using knockdown hiPSCs with 31.33% LSD1 activity, we achieved a four-step differentiation into IPCs and test its differentiation efficiency, morphology, and marker genes and proteins. We implanted the IPCs into the renal subcapsular of SCID-Beige diabetic mice to evaluate the hypoglycemic effect in vivo. We tested LSD1 and HDAC1 whether they are present in the CoREST complex through IP-WB, and analyzed LSD1, CoREST, HDAC1, H3K4me2/me3, and H3K27me3 protein expression before and after knockdown of LSD1.

**Results:**

Differentiated hiPSCs were 38.32% ± 3.54% insulin-positive cells and released insulin/C-peptide in response to glucose stimulus in a manner comparable to adult human islets. Most of the IPCs co-expressed mature β cell-specific markers. When transplanted under the left renal capsule of SCID-Beige diabetic mice, these IPCs reversed hyperglycemia, leading to a significant increase in the definitive endoderm cells. IP-WB results showed that LSD1, HDAC1, and CoREST formed a complex in hiPSCs. Chip-PCR results showed that LSD1, HDAC1, and CoREST were enriched in the same district during the SOX17 and FOXA2 promoter region. Inhibition of LSD1 would not affect the level of CoREST but decreased the HDAC1 expressions. The H3K4me2/me3 and H3K9act level of SOX17 and FOXA2 promoter region increased after inhibited of LSD1, and promoted transcriptional activation. The H3K4me2/me3 and H3K9act level of OCT4 and SOX2 promoter region decreased with the transcriptional repressed.

**Conclusions:**

LSD1 regulated histone methylation and acetylation in promoter regions of pluripotent or endodermal genes. Our results suggest a highly efficient approach to producing IPCs from hiPSCs.

## Introduction

Similar to embryonic stem cells (ESCs), newly developed induced pluripotent stem cells (iPSCs) can be proliferated and differentiated to specific cell types [1, 2], indicating a promising approach to generating anti-diabetic drugs and insulin-producing cells (IPCs). In addition, iPSCs share the same genetic information as the donor cells and therefore should be recognized as “self” by the donor’s immune system, suggesting the exciting potential for autologous therapy with patient-derived iPSCs that may eliminate the need for immunosuppression. Most importantly, the use of human iPSCs (hiPSCs) avoids the ethical issues surrounding the use of ESCs. Recently, groups around the world have reported different protocols to induce IPCs from ESCs or iPSCs. Rezania et al. established a 7-stage protocol that induces human ESCs into IPCs in 15 days [3]. Stage (S) 7 cells express key markers of mature pancreatic β cells, including MAFA, and display glucose-stimulated insulin secretion similar to that of human islets. Pagliuca et al. reported a strategy for the production of glucose-responsive IPCs from human PSCs via an 8-stage sequential modulation of multiple signaling pathways in 35 days; these cells mimicked the function of human islets both in vitro and in vivo [4]. Although insulin-producing β cells derived from ESCs/iPSCs have been successfully induced and multiple signaling pathways involved in the differentiation of ESCs/iPSCs into IPCs have been reported, much work remains to be done to achieve mature β cells. Our previous study found that lysine-specific demethylase 1 (LSD1) is an important factor to maintain the balance between the self-renewal and differentiation of hiPSCs. We used LSD1 inhibitors and RNAi technique to establish hiPSCs with different LSD1 activity. When LSD1 activity is low, the self-renewal capacity of hiPSCs is repressed, but the differentiation process is activated. The point of LSD1 activity at 50% may serve as a switch that hiPSCs turn to differentiate [1]. Here, we propose a new strategy to acquire IPCs from hiPSCs by knocking down LSD1 on the basis of a four-stage protocol [5]. We generated hiPSCs with different LSD1 activities and then determined an optimum activity for redirecting hiPSCs from proliferation to differentiation that can be applied to induce the highly efficient differentiation of hiPSCs into human IPCs with substantial maturity. Furthermore, we transplanted human IPCs into immunodeficient diabetic mice to observe their ability to reverse hyperglycemia. Finally, we explored the mechanism of LSD1 in regulating epigenetic changes in hiPSCs that are associated with the highly efficient differentiation to IPCs. Our results suggest an efficient approach to producing autologous IPCs with high potential for therapeutic benefit.

## Experimental section

### hiPSCs and culture

hiPSCs (NF1-4-iPS-C11, UMC1, and UMC6) were generous gifts from the Guangzhou Institute of Bioscience and Health, Chinese Academy of Sciences [6]. They were generated from human skin fibroblast cells that were transduced with a cocktail of BMX-based retroviruses encoding human SOX2, Klf4, OCT4, and c-Myc (Addgene, Cambridge, MA, USA). hiPSCs were maintained on Matrigel (BD Biosciences, Bedford, MA, USA) in mTeSR™1 (Stemcell Technologies, Vancouver, BC, Canada) without a feeder culture system. The medium was changed daily, and the cells were passaged when confluency reached 80–90%.

### Knockdown of LSD1 in hiPSCs with shRNA

Five shRNA plasmids (scrambled-shRNA, shRNA-LSD1-2495, shRNA-LSD1-863, shRNA-LSD1-927, and shRNA-LSD1-1086) constructed with a pGLV2-U6-Puro vector were delivered into hiPSCs via lentiviral transduction. After 24 h, we used positive clones selected with puromycin for 72 h and then quantified LSD1 expression by real-time PCR. On the basis of mRNA expression, shRNA-LSD1-927 showed the highest inhibition effect of LSD1.

### Assessment of the effects of shRNA on hiPSC proliferation, cell cycle, and apoptosis

shRNA-treated hiPSCs were cultured in an incubator at 37 °C for 4 h after adding 10 μL of CCK-8 (WST-8, Dojindo, Japan). Absorbance was tested at 450 nm by a multiscan spectrum (Bio-Rad Model 680, USA). For cell cycle analysis, cells were fixed in 70% ethanol overnight, stained with 2 μL of PI (50 mg/mL; KeyGEN Biotech), and kept in the dark for 15 min at room temperature. Apoptosis assays were carried out using the Annexin V-FITC Apoptosis Detection Kit (KeyGEN Biotech). FITC-Annexin V (5 μL) and PI (5 μL) were added to the treated cells for nuclear staining and then incubated at room temperature for 30 min. The apoptosis rate and cell cycle were finally analyzed by flow cytometry (BD Biosciences, USA).

### RT-PCR and quantitative PCR analysis of gene expression

RT-PCR and qRT-PCR were performed as previously described [7]. Total RNA was isolated using TRIzol reagent (Takara Bio, Inc., Otsu, Japan) and then reverse-transcribed into cDNA using a PrimeScript RT Reagent kit with gDNA eraser (Takara) following the manufacturer’s instructions. Quantitative PCR was performed on a multicolor real-time PCR detection system (Bio-Rad Laboratories) by using SYBR Green PCR Master Mix (Toyobo, Osaka, Japan). The primer sequences used for qPCR and product size are shown in Table S1 (Supporting Information). The amplification conditions consisted of an initial denaturation step at 95 °C for 15 min, followed by 40 cycles of denaturation at 94 °C for 15 s, annealing at the designated temperature for 30 s, and extension at 72 °C for 30 s.

### Culture and differentiation of hiPSCs into pancreatic β cells

After transduction with scrambled-shRNA, shRNA-LSD1-927-treated hiPSCs were treated with puromycin for 72 h. A modified four-step induction protocol to direct pancreatic differentiation from hiPSCs was employed as described previously [5]. In stage 1 of the procedure, the hiPSCs were cultured for 4 days to form definitive endoderm in DMEM/F12 containing 0.2% BSA (Sigma, USA), 0.5× N2-supplement (Gibco, USA), 0.5× B-27 supplement (Gibco, USA), 100 ng/mL Activin A (Peprotech, USA), and 1 μM Wortmannin (Sigma, USA) with medium changes every day. In stage 2, the differentiated cells were cultured for 4 days in F12/IMDM (1:1) mixed with 0.5% BSA, 0.5× ITS-X supplement (Gibco, USA), 0.5× B-27 supplement, 2 μM RA (Sigma, USA), 20 ng/mL FGF7 (Peprotech, USA), and 50 ng/mL Noggin (Peprotech, USA) with medium changes every day. In stage 3, the cells were cultured for 5 days in DMEM/H (high glucose) containing 0.5% BSA, 1× ITS-X supplement, 1× N2-supplement, and 50 ng/mL EGF (Sigma, USA). The medium was refreshed every day. For maturation, cells were incubated another 7–9 days in DMEM/F12 containing 1× ITS-X supplement, 10 ng/mL bFGF (Peprotech, USA), 10 mM nicotinamide (Sigma, USA), 50 ng/mL Exendin-4 (Sigma, USA), and 10 ng/mL BMP4 (Peprotech, USA). All media and supplements were purchased from Gibco, and growth factors were from Peprotech, unless indicated.

### Morphology and dithizone staining

Morphological changes at different stages of hiPSC differentiation were observed by microscopy. Subsequently, 21-day cell clusters were washed twice with PBS (pH 7.4) and incubated with 1 mL of dithizone (DTZ) solution at 37 °C for 15 min. An inverted microscope was used for the observation of staining.

### Immunofluorescence assay

The sample cells were fixed in 4% paraformaldehyde for 10 min, washed with PBS, permeabilized with 0.3% Triton X-100 for 10 min, and then blocked with 5% BSA at 37 °C for 1 h. Then, they were incubated with primary antibody at 37 °C for 1 h and further incubated with secondary antibody for 1 h. The primary antibodies were as follows: rabbit anti-human insulin (1:200, Abcam), goat anti-human C-peptide (1:50, R&D Systems), goat anti-human PDX1, rabbit anti-human NKX6.1, and goat anti-human Glucagon (1:50, Santa Cruz). The secondary antibodies were FITC-conjugated goat anti-rabbit IgG (Jackson Lab) and Rhodamine Red-conjugated donkey anti-goat IgG Rhodamine Red antibody (Invitrogen-Biosource). After washing, DAPI (10 μg/mL) was added to stain the nuclei at room temperature for 8 min. After adding an anti-fade mounting medium, images were captured using a fluorescent microscope (Eclipse TE2000-U; Nikon, Düsseldorf, Germany).

### Insulin/C-peptide release assay by ELISA

Adult human islets were isolated according to a standard procedure by our group [8]. hiPSC-derived IPCs and adult human islets were incubated with Krebs Ringer Buffer supplemented with 30 mM KCl and 2.5 mM (L) or 27.5 mM (H) glucose at 37 °C for 2 h. The release level of insulin and C-peptide was detected by using a human insulin or C-peptide ELISA kit (Mercodia, USA).

### Preparation of diabetic mice and cell transplantation

Eight-week-old SCID-Beige mice (weighing 20–25 g) were intraperitoneally injected with 2% streptozotocin at a dose of 170 mg/kg of body weight. Blood glucose was measured with an Accu-Chek glucose meter (Roche). Mice showing blood glucose levels over 300 mg/dL for 3 consecutive days were considered diabetic and used for cell transplantation. Diabetic mice were randomly divided into four groups: IPCs (*n* = 8)—1 × 10^7^ routinely induced IPCs-scrambled-shRNA were transplanted into the left renal capsule; IPCs-shRNA-LSD1-927 (*n* = 8)—after LSD1 knockdown with shRNA-LSD1-927, 1 × 10^7^ hiPSC-derived IPCs were transplanted into the left renal capsule; adult human islets (*n* = 8)—approximately 500–600 adult islet equivalents containing 1 × 10^7^ pancreatic β cells were transplanted into the left renal capsule; and diabetes (*n* = 6)—0.1 mL of PBS was injected into the left renal capsule. Six other normal SCID-Beige mice were set as a negative control. The project was approved by the Second Clinical Medical College Ethical Committee (Shenzhen People’s Hospital), Ji’nan University.

### Function detection and pathological observation after transplantation

Fasting blood glucose was measured every 3 days after transplantation. Each time, five mice were randomly selected. If the fasting blood glucose was lower than 250 mg/dL twice, the blood glucose level was defined as normal. On days 7, 14, 21, and 28 after cell transplantation, 0.5 mL of whole blood was collected from the orbit, and the serum was separated by centrifugation and stored at − 20 °C for blood insulin detection. On days 7 and 21, after fasting for 6–8 h, mice were intraperitoneally injected with 10% glucose solution for glucose tolerance testing. Left kidneys were removed in 28 days and embedded in paraffin. HE staining and immunofluorescence staining were performed to observe cell survival and insulin and glucagon expression. On day 30 after transplantation, the mice were sacrificed.

### Immunoprecipitation-Western blot

In accordance with the protocol by Hattori [9], after the first stage of induction, shRNA-LSD1-927-treated hiPSCs were lysed with RIPA lysis buffer. Approximately 1000 μg of proteins was diluted with NET-gelatin buffer. Subsequently, rabbit anti-human LSD1, HDAC1, and CoREST antibodies (0.5–1 μg, Active Motif, USA) were added to the sample. An equal amount of normal rabbit serum was then added to the control group. After incubation with Agarose-A/G (sc2003, Santa Cruz, USA; 30 mL) on a 40 °C shaker overnight, precipitates were collected and washed three times. After the addition of 2× loading buffer and heat denaturation, proteins were separated on an SDS-PAGE gel. The primary antibodies used were as follows: mouse anti-human LSD1, HDAC1, and CoREST monoclonal antibody (1:200, Active Motif, USA). HRP-labeled horse anti-mouse IgG (1:200, L0807, Vector Laboratories, USA) was used for DAB staining.

### Chromatin immunoprecipitations

Chromatin immunoprecipitation (ChIP) analysis was performed as reported previously [9]. RNAi- or LSD1 inhibitor-treated hiPSCs were exposed to 1% formaldehyde to cross-link the proteins, and 2.0 × 10^6^ cells were used for each ChIP assay. After the DNA was sonicated into 200–1000 bp fragments, target antibodies were added overnight at 4 °C. The antibodies against H3K4me2/me3 (dimethylation and trimethylation), H3K9ac (H3K9 acetylation), and H3K9me2/me3 were purchased from Millipore, USA. Quantitative ChIP was performed by using qPCR on the real-time PCR detection system using the enzyme master mix from Takara. Pluripotent genes OCT4 and NANOG and endoderm differentiation genes SOX17 and FOXA2 were tested by qPCR. The primer sequences used for qPCR for ChIP are shown in Table S2 (Supporting Information).

### Statistical analyses

In all cases, values are presented as mean ± standard deviation. Student’s *t* test and one-way ANOVA were used to compare differences between different groups. Statistical analysis was performed using SPSS version 16.0 (SPSS, Chicago, IL, USA). Differences between groups were considered significant when *P < 0.05 and **P < 0.01.

## Results

### Effects of LSD1 on hiPSC self-renewal

We found that hiPSCs-shRNA-LSD1-927 reduced the LSD1 activity to only 31.33% ± 4.03% of the hiPSCs-scrambled-shRNA (*P* < 0.01). Western blot analysis for LSD1 protein and qPCR analysis for LSD1 mRNA yielded similar results as that of the LSD1 activity test. We then monitored the proliferation of hiPSCs-shRNA-LSD1-927 by using a CCK-8 assay (Fig. S1A). The growth rate of shRNA-LSD1-927-hiPSCs was slower than that of hiPSCs-scrambled-shRNA (*P* < 0.01). These results indicate that LSD1 activity may be associated with hiPSC proliferation ability. We did not observe the same trend of LSD1 activity on cell apoptosis. The apoptosis rate of hiPSCs-shRNA-LSD1-927 (2.3% ± 0.56%) and hiPSCs-scrambled-shRNA (2.3% ± 0.43%) did not change (*P* > 0.05, Fig. S1B). Flow cytometry was performed to explore the effects of LSD1 activity on the cell cycle. hiPSCs-shRNA-LSD1-927 was arrested (46.3% ± 1.63%) more than hiPSCs-scrambled-shRNA (21.4% ± 1.63%) in the G0/G1 phase (*P* < 0.01, Fig. S1C and S1D). The above data indicate that LSD1 plays a key role in regulating hiPSC self-renewal by influencing cell proliferation but has no influence on cell apoptosis.

### Effects of LSD1 on hiPSC pluripotency and differentiation genes

To observe the morphologic changes of hiPSCs-shRNA-LSD1-927 clones after LSD1 knockdown, we performed microscopy. After 72 h, the cell colonies from the control group (hiPSCs-scrambled-shRNA) were oval with smooth edges, suggesting typical ESC morphology. The morphology of the hiPSCs-shRNA-LSD1-927 colonies showed significant changes: cells became much bigger and flattened, with an increased proportion of cytoplasm and many dispersed cells around the colony’s edge. After 2–3 passages, hiPSCs-shRNA-LSD1-927 no longer formed intact colonies but grew separately as dispersed single cells (Fig. S2A). This result indicates that the modulation of LSD1 activity affects the morphology of hiPSCs, increasing their ability to differentiate as LSD1 activity is decreased.

To further examine the effect of reduced LSD1 activity on differentiation, we performed qRT-PCR analysis of the expression of pluripotent and developmental genes in hiPSCs 72 h after reducing LSD1 activity with hiPSCs-shRNA-LSD1-927. When LSD1 activity was knocked down with shRNA, the expression of pluripotency genes OCT4, SOX2, and NANOG decreased significantly (*P* < 0.05). This decrease was the most significant for cells treated with shRNA-LSD1-927. However, the endodermal gene SOX17 increased 32 times and FOXA2 increased 19 times in hiPSCs-shRNA-LSD-927 compared with the hiPSCs-scrambled-shRNA (*P* < 0.01, Fig. S2B). The expression of TUBB3, an ectodermal marker gene, remained stable. These results suggest that the proliferation of hiPSCs was significantly decreased, and the ability to differentiate was significantly enhanced when LSD1 activity was reduced to less than 50%.

### hiPSCs-shRNA-LSD1-927 can be differentiated into IPCs that express islet cell-specific markers

**Fig. 1:**
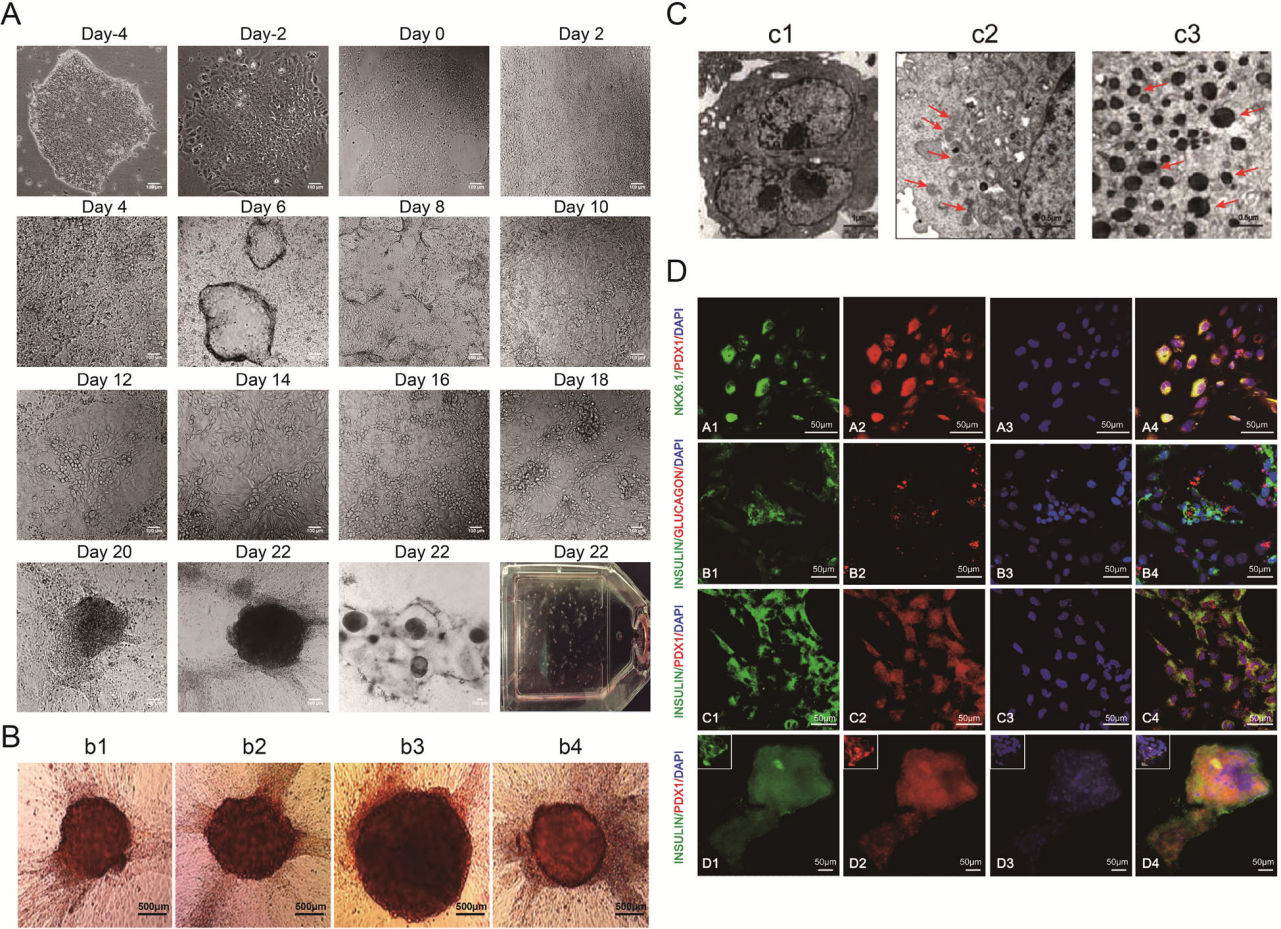
Morphology and phenotype characteristics of pancreatic β cells derived from hiPSCs at the final maturation stage. **A** Morphological changes of hiPSCs during differentiation into mature pancreatic β cells. **B** The pancreatic β cells derived from hiPSCs were stained with DTZ. Scale bars, 500 μm (b1, b2, b3, b4). **C** Scanning electron microscopy of IPCs derived from hiPSCs. The IPCs have secretory granules and complete capsules. Scale bars, 1 μm (c1); 0.5 μm (c2, c3). **D** Immunofluorescence staining showing that the differentiated hiPSCs at the final mature stage co-expressed PDX1 and NKX6.1, insulin and glucagon, and PDX1 and insulin (scale bars = 50 μm)

To assess the potential of hiPSCs with reduced LSD1 activity to differentiate into IPCs, we developed a highly efficient 4-step protocol. On day 2 after hiPSCs-shRNA-LSD1-927 transduction, the colony edge started to lose its intactness and became dissociated. The cells increased in size, and nuclei became small, indicating that the cells had started to differentiate. After puromycin screening for 48 h, non-transduced cells were removed. We then started the 4-step induction process. On day 2 of this process, almost all of the cell units merged together. On day 4, the cells started to form three-dimensional structures. On day 6, abundant three-dimensional physalis was apparent. On day 8, vacuoles collapsed and became flattened. On day 10, cells proliferated as notochord-like structures in the collapsed physalis. On day 12, the notochord-like cells formed clusters. On day 14, the notochord-like cell clusters in the collapsed vacuoles merged together. On day 16, the cells in the cluster became spherical and grew to the cluster center. On day 18, the cell clusters started to form a cell mass. On day 20, the cell mass became bigger. Finally, on day 22, many cell masses could be observed in the flask (Fig. 1A).

The differentiated IPCs presented as dense cell masses or spherical structures. To determine whether they could express insulin, we performed staining assays with DTZ, an agent that specifically stains insulin granules in β cells [8]. The clusters were DTZ-positive, whereas undifferentiated cells (cells not grown in clusters) were DTZ-negative in the absence of glucose (Fig. 1B). Electron microscopy showed that in the absence of glucose stimulation, no granules formed in the cytoplasm of the differentiated IPCs. However, when the cells were incubated with 27.5 mM glucose for 15 min, naïve insulin granules formed in the Golgi apparatus. After stimulation with 27.5 mM glucose for 2 h, mature insulin granules were abundant in the cytoplasm of differentiated IPCs (Fig. 1C). These results indicate that induced IPCs can secrete insulin in response to glucose. During the final stage of our approach, the population of PDX1-positive cells was observed to be robust. Many PDX1-positive cells co-expressed NKX6.1 or insulin [9]. We also observed that these differentiated cells contained both insulin- and glucagon-positive cells (Fig. 1D), which confirmed that these differentiated cells included both pancreatic β and α cells that could express insulin and glucagon.

**Fig. 2:**
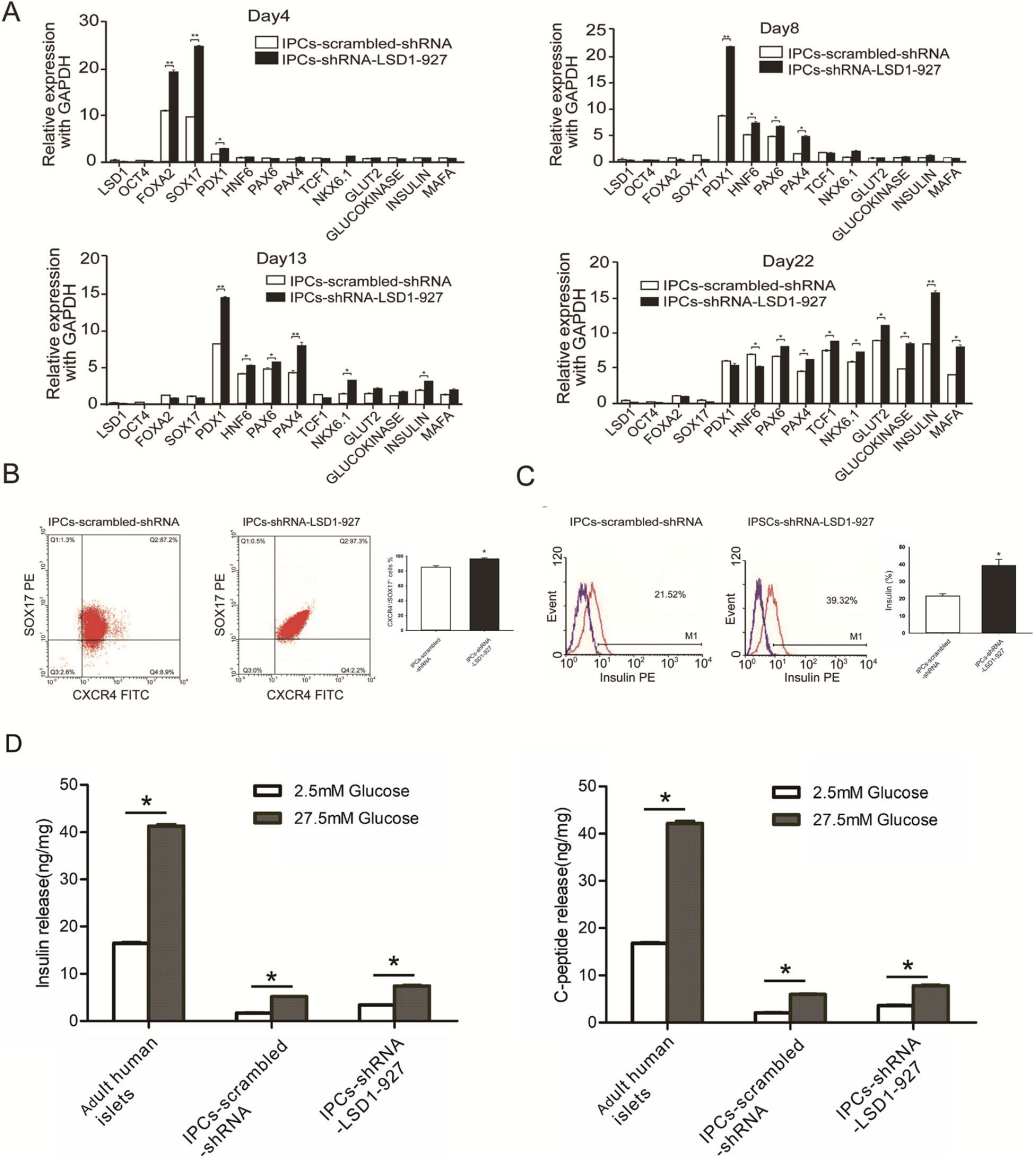
Functional characteristics in the pancreatic β cells derived from hiPSCs at the final maturation stage. **a** Gene expression of pancreas-related developmental genes during hiPSC differentiation to IPCs (*n* = 3). **b** Flow cytometry analysis of differentiated hiPSCs revealed that after stage 1, 96.3% ± 1.54% of the cells with shRNA-LSD1-927 were SOX17^+^ and CXCR4^+^ double-positive, and 85.06% ± 2.14% of the control cells (scrambled-shRNA) were double-positive. A significant difference was observed between the two groups (*P < 0.05, *n* = 3). **c** Flow cytometry analysis revealed that IPCs at the final maturation stage derived from hiPSCs-shRNA-LSD-927 were 38.32% ± 3.54% and those derived from hiPSCs-scrambled-shRNA were 20.42% ± 1.36%. A significant difference was found between the two groups (*P < 0.05, *n* = 3). **d** Differentiated IPCs release insulin and C-peptide (*n* = 3). Glucose-induced insulin release data correspond to the amount of insulin and C-peptide secreted for 1 h after 2.5 and 27.5 mM glucose stimulation

To further characterize the IPCs-scrambled-shRNA and IPCs-shRNA-LSD1-927 at the final stage, we examined their gene expression at the mRNA level. At induction stage 1 (day 4), SOX17, FOXA2, and PDX1 gene expression was high. At stage 2 (day 8), PDX11, HNF6, PAX4, and NKX6.1 were upregulated, whereas SOX17 and FOXA2 were downregulated. At stage 3 (day 13), PAX4, HNF6, PAX6, and NKX6.1 were further upregulated, and insulin mRNA expression began to rise. At stage 4 (days 22), the expression levels of PDX1, HNF6, PAX6, PAX4, TCF1, NKX6.1, glucokinase, insulin, and MAFA were high in the induced IPCs. The expression of these pancreatic genes was higher in IPCs-shRNA-LSD1-927 cells compared with that in IPCs-scrambled-shRNA (*P* < 0.05, Fig. 2a), indicating that our approach resulted in high differentiation efficiency. Moreover, the expression dynamics of most specific β cell marker genes during IPC differentiation from hiPSCs was similar to that of pancreatic β cell development in vivo [10, 11].

To further assess whether the hiPSCs following transfection with scrambled-shRNA and shRNA-LSD1-927 formed definitive endoderm, we examined the co-expression marker of CXCR4 and SOX17 [12]. More CXCR4 and SOX17 double-positive cells were observed at day 4 in hiPSCs-shRNA-LSD1-927 cultures (96.3% ± 1.54%) than in hiPSCs-scrambled-shRNA cultures (85.06% ± 2.14%) (*P* < 0.05, Fig. 2b). These results indicate that LSD1 downregulation by shRNA-LSD1-927 significantly increased the differentiation efficiency to endoderm. After day 21 of induction, the percentage of insulin positivity reached 38.32% ± 3.54% for IPCs-shRNA-LSD1-927 and only 20.42% ± 1.36% for the control IPCs-scrambled-shRNA (Fig. 2c). Compared with the previously reported [3, 4, 13] efficiency of 7.3% or 25% for insulin/C-peptide-positive cells as assayed by flow cytometry analysis, our approach resulted in higher efficiency. On the basis of these findings, we further analyzed the potential of glucose-stimulated C-peptide release, which is one of the important functional indices in β cells. The insulin and C-peptide release with high glucose stimulation were significantly higher than those with low glucose, which indicated that the differentiated cells secreted insulin and C-peptide in response to high glucose stimulation. Furthermore, the level of glucose-regulated insulin and C-peptide released by IPCs-shRNA-LSD1-927 was greater than that of IPCs-scrambled-shRNA (*P* < 0.05, Fig. 2d), though the release was still much less efficient than that of adult human islets (approximately 1/8 as much insulin and 1/6 as much C-peptide as for adult human islets).

### Reversal of hyperglycemia after IPC transplantation

**Fig. 3:**
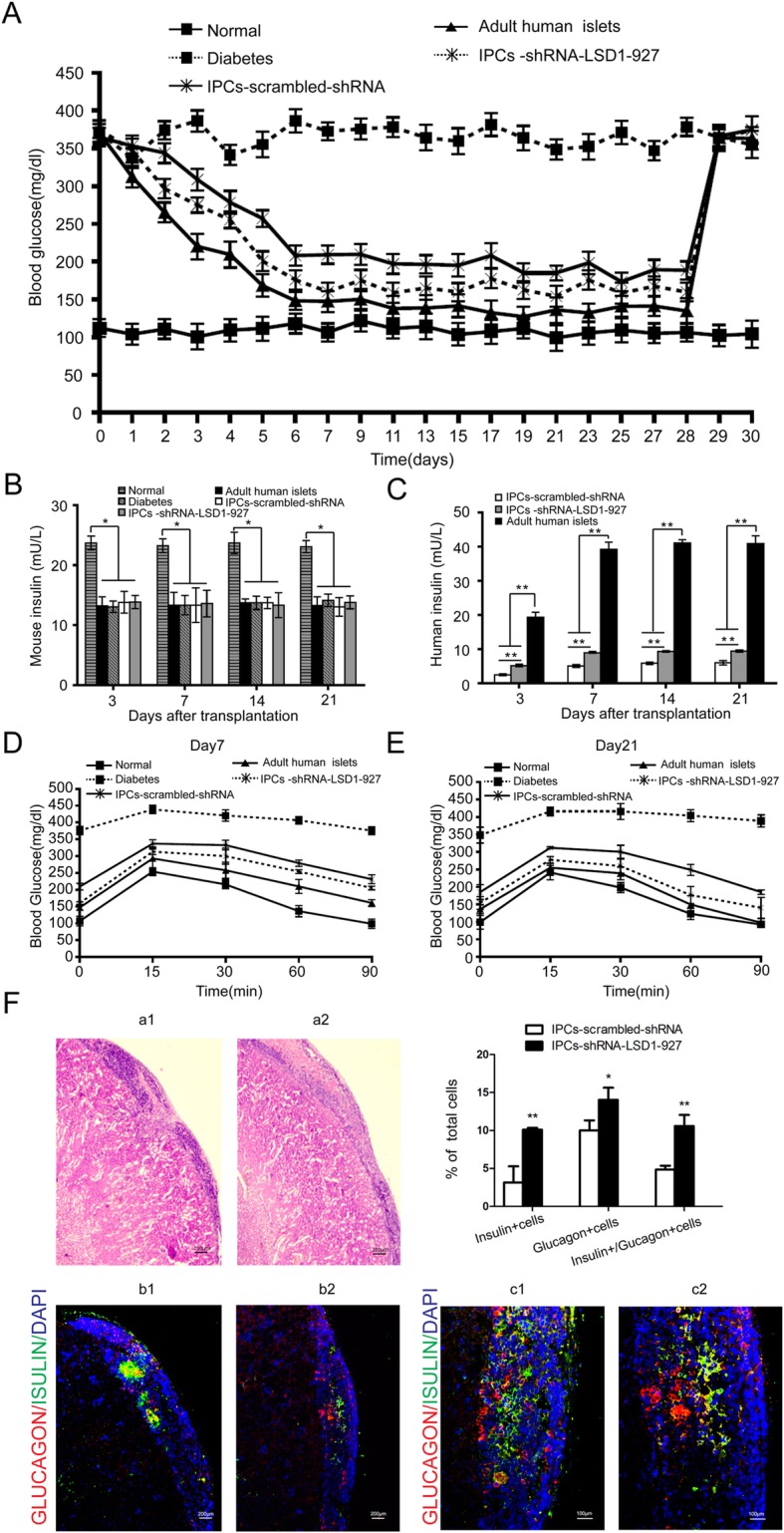
Function detection and pathological observation after cell transplantation. **a** Glucose levels were monitored every 3 days after transplantation (*n* = 8). The left kidney was removed 28 days after transplantation. Data presented are means ± SD. **b**, **c** Human insulin and mouse insulin levels were detected in mice after transplantation (*n* = 8). Mouse insulin levels were not changed after transplantation, and human insulin levels significantly increased. **d**, **e** Blood glucose levels during a glucose tolerance test (*n* = 3). The blood glucose curves are shown at 7 and 21 days after IPC transplantation. **f** Immunofluorescence staining showed insulin (green) and glucagon (red) double-positive cells derived from renal tissue. The results suggest that transplanted IPCs surviving under the left renal subcapsular (a1, a2) can secrete insulin and glucagon, which is consistent with this cell population including pancreatic β and α cells. In addition, the expression of insulin- and glucagon-positive cells from IPCs-shRNA-LSD1-927 (b1, c1) was higher than that of the IPCs-scrambled-shRNA group (b2, c2). Scale bars, 200 μm (b1, b2); 100 μm (c1, c2). (*P < 0.05; **P < 0.01, *n* = 3)

We transplanted differentiated IPCs-shRNA-LSD1-927, IPCs-scrambled-shRNA, and adult human islets under the left renal capsule of diabetic SCID-Beige mice. On day 3 after transplantation, blood glucose levels in all of the groups, especially IPCs-shRNA-LSD1-927, started to drop quickly. However, the anti-hyperglycemic effects of these two IPCs did not reach the level of adult human islets. On day 28, the blood glucose levels immediately increased after the removal of the left renal capsule, indicating that the transplanted cells resulted in glucose homeostasis (Fig. 3a). Sera were collected from all of the groups at different time points and analyzed using human and mouse insulin ELISA kits. Mouse insulin levels in all of the transplanted groups were lower than those in normal mice at all time points (*P* < 0.05). No difference was observed compared with diabetic mice (*P* > 0.05, Fig. 3b). However, human insulin levels in the IPCs-shRNA-LSD1-927 group were significantly higher than those of the control IPCs-scrambled-shRNA group (*P* < 0.05), which were much lower than those in the adult human islet group (*P* < 0.05, Fig. 3c). This result indicated that the IPCs-shRNA-LSD1-927 released human insulin. Glucose tolerance tests were performed on days 7 and 21 after transplantation. Blood glucose levels in the IPCs-shRNA-LSD1-927 group were lower than those in the IPCs-scrambled-shRNA group 15 min after glucose stimulation. This finding indicates that IPCs-shRNA-LSD1-927 caused superior glucose control and tolerance compared with the controls, though the level was still much lower than that for adult human islets (Fig. 3d, e). Finally, to examine the histopathologic effects of transplantation, on day 28, we removed the left kidneys from SCID-Beige mice. Numerous surviving cells could be observed under the capsule in IPCs-shRNA-LSD1-927 and IPCs-scrambled-shRNA mice. Immunostaining showed that surviving cells still expressed insulin (green) and glucagon (red), indicating that transplanted IPCs could function as β and α cells. Furthermore, the IPCs-shRNA-LSD1-927 had more insulin- and glucagon-positive cells than IPCs-scrambled-shRNA (*P* < 0.05, Fig. 3f).

### Mechanism of LSD1 in regulating the efficiency of hiPSC differentiation to IPCs through the modulation of promoter interactions

**Fig. 4:**
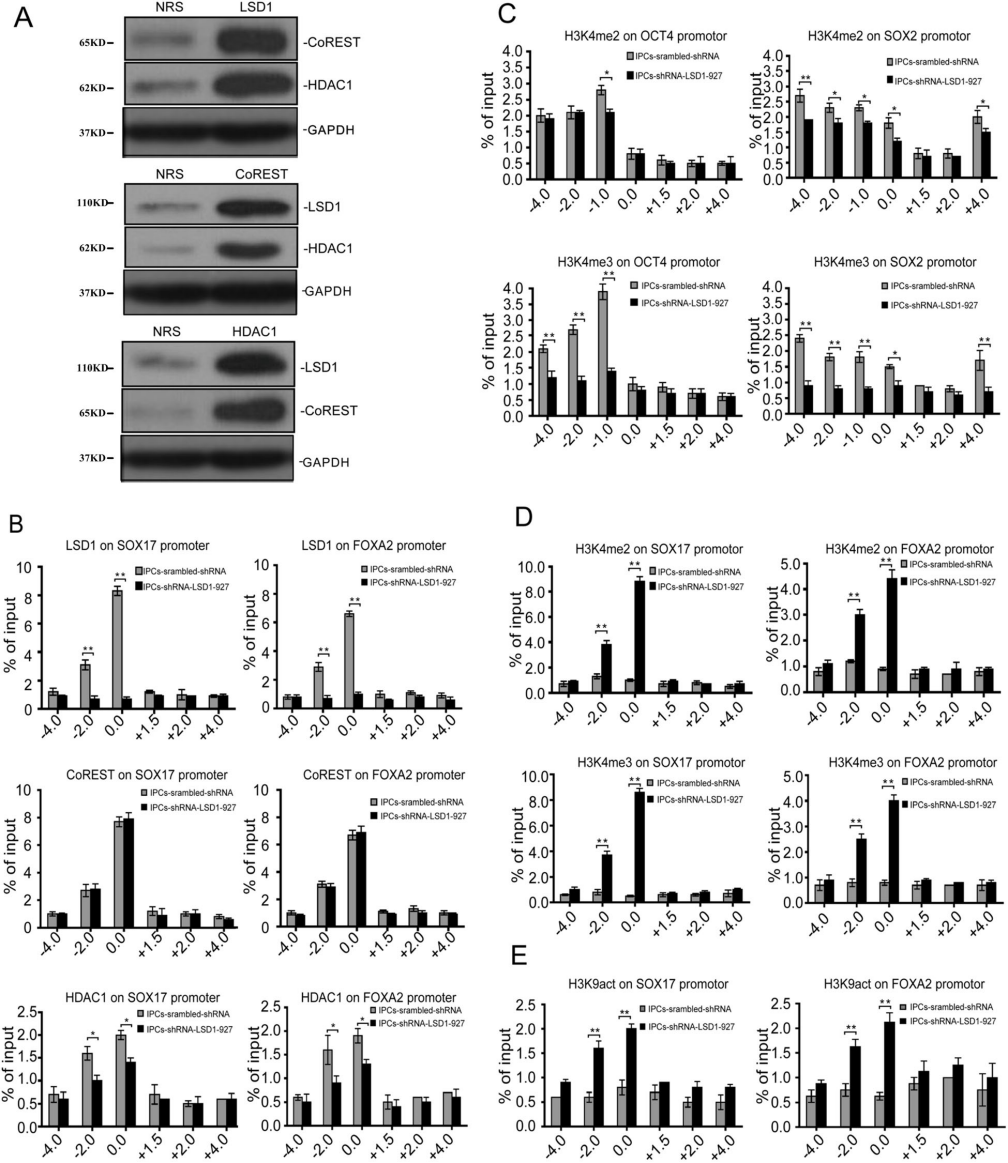
IP-WB and ChIP-qPCR assessment of the effects of LSD1 knockdown on promoter complexes. **a** LSD1 and HDACI coexistence with the CoREST complexes was confirmed with IP-WB (NRS as normal rabbit serum, control group). **b** After shRNA-LSD1-927 knockdown, LSD1/CoREST/HDAC1 complexes in the SOX17/FOXA2 gene promoter regions show differences. **c** After shRNA-LSD1-927 knockdown, the histone modification levels of H3K4me2/me3 in the OCT4/SOX2 gene promoter regions show differences. **d** After shRNA-LSD1-927 knockdown, the histone modification levels of H3K4me2/me3 in the SOX17/FOXA2 gene promoter regions show differences. **e** After shRNA-LSD1-927 knockdown, the histone modification levels of H3K9act in the SOX17/FOXA2 gene promoter regions show differences (*P < 0.05; **P < 0.01, *n* = 3)

To assess the mechanism of LSD1 in promoting IPC production, we confirmed whether LSD1 and HDAC1 exist within the CoREST complex (Fig. 4a) [14]. The amplification of the fragments captured by LSD1, HDAC1, and CoREST antibodies, which flank regions upstream and downstream of SOX17 and FOXA2 promoters, showed that these proteins all reside 2000 and 100 bp upstream of the transcription start sites (TSS) of SOX17 and FOXA2, respectively. For LSD1 knockdown by shRNA-LSD1-927, the amounts of LSD1 and HDAC1 that occupied these promoter regions of SOX17 and FOXA2 were obviously less (*P* < 0.01, *P* < 0.05), but CoREST was unchanged (*P* > 0.05, Fig. 4b). To demonstrate that the downregulation of OCT4 and SOX2 was related to H3K4 methylation levels in the promoter regions, we designed primers targeting upstream and downstream (approximately − 4000, − 2000, − 1000, − 100, + 1000, + 2000, and + 4000) to amplify the segments captured by H3K4 me2/me3. When LSD1 was inhibited, the dimethylation level of H3K4 in the promoter region 1000 bp upstream of OCT4 TSS decreased (*P* < 0.05), and the trimethylation level of H3K4 in the region 4000, 2000, and 1000 bp upstream of OCT4 TSS decreased dramatically (*P* < 0.01). For the SOX2 promoter region, we observed a similar phenomenon, wherein the dimethylation and trimethylation levels of H3K4 in the promoter region and upstream regions of the SOX2 TSS were significantly downregulated (*P* < 0.05, *P* < 0.01; Fig. 4c). For SOX17 and FOXA2, we designed primers targeting the region at approximately − 4000, − 2000, and − 100 bp and + 1500, + 2000, and + 4000 bp of the TSS to amplify the segments captured by H3K4 me2/me3. H3K4 me2/me3 was mostly observed in the region where the LSD1 resides, which was at approximately 2000 and 100 bp upstream of SOX17 and FOXA2 TSS, respectively (Fig. 4d). This finding indicates that LSD1 does not directly reverse H3K4 trimethylation but can regulate the dimethylation level of H3K4 and finally influence the trimethylation level of H3K4 [15]. In hiPSCs, LSD1 and HDAC1 were detected in the promoter region at approximately 2000 and 100 bp upstream of the SOX17 and FOXA2 TSS, respectively. However, the acetylation of H3K9 (H3K9act) could not be recruited to the same region. After LSD1 was inhibited, H3K9act was significantly upregulated in the region, which could explain the transcriptional activation of SOX17 and FOXA2 (Fig. 4e).

## Discussion

The histone demethylase LSD1 plays a key role in hiPSC proliferation and differentiation in our study. The hiPSCs were differentiated into endodermal tissue when shRNA-LSD1-927 reduced the LSD1 activity to only 31.33% of the control. Either inhibition of LSD1 activity or knockdown of LSD1 expression led to dramatically reduced hiPSC proliferation and increased hiPSC differentiation [16]. LSD1 is also essential for enhancers to be decommissioned during the differentiation of mouse ESCs. It occupies the enhancers of pluripotent genes (OCT4, SOX2, and NANOG) that are critical for the control of the pluripotent state of ESCs. Therefore, decreased LSD1 expression results in the silencing of these pluripotent genes and the differentiation of ESCs [17]. In general, the expression level of LSD1 is high in undifferentiated human ESCs and is progressively downregulated during differentiation. LSD1 knockdown in human ESCs by RNAi results in the downregulation of pluripotent genes and the upregulation of endomesoderm developmental genes (FOXA2, EOMES, BMP2, and SOX17), which finally turns on the differentiation process [18]. Further studies show that LSD1 binds to OCT4 and NANOG promoters through its REST domain. With LSD1 knockdown, H3K4 methylation levels in the promoter regions of these target genes are upregulated, and differentiation genes are activated, which serves as a hallmark of the loss of ESCs’ self-renewal ability and the start of differentiation [15]. These findings indicate that LSD1 plays an important role in hiPSCs’ self-renewal and differentiation. OCT4 is a key transcription factor in the silencing of the expression of developmental genes in hiPSCs [19, 20] and can interact with LSD1 [21]. Once pluripotent genes are inhibited, hiPSCs lose their self-renewal ability and turn on their differentiation program to three germ layers. We hypothesize that LSD1 may regulate pluripotent gene expression in an indirect way and may play a key role in the balance between self-renewal and differentiation of hiPSCs.

We silenced LSD1 in hiPSCs with shRNA-LSD1-927 and directed these cells to differentiate into IPCs by a four-step induction process (Fig. 1A). Moreover, these cells were positive for DTZ staining (Fig. 1B) and co-expressed pancreas-specific transcription factors PDX1 and NKX6.1 with insulin. Apart from β cell markers, the α cell marker glucagon was found in the same cells expressing insulin, indicating that IPCs have the characteristics of both α cells and β cells (Fig. 1D). TEM analysis demonstrated that insulin-secreting granules formed immediately in the cytoplasm of IPCs derived from hiPSCs with LSD1 knockdown by shRNA-LSD1-927 after stimulating with high glucose, indicating that these differentiated IPCs have the potential to respond to glucose changes and secrete insulin in a glucose-regulated manner (Fig. 1C). At the end of the first stage, SOX17 expression in shRNA-LSD1-927-treated hiPSCs was 2.6 times that of control hiPSCs. Flow cytometry results showed that the endoderm differentiation efficiency (SOX17^+^CXCR4^+^ is the surface marker on defining endoderm cells) increased by approximately 10% (Fig. 2a, b). SOX17 activates the differentiation process of primitive and definitive endoderm during embryonic development [22, 23]. In addition, it induces ESCs to differentiate into primitive endoderm [24], especially body wall endoderm [25, 26]. Therefore, we hypothesize that the loss of LSD1 may activate SOX17 and promote hiPSCs to differentiate into endoderm.

C-peptide expression was used to assess the differentiation efficiency. The differentiation efficiency of control hiPSCs was 20.42% ± 1.36% and increased to 38.32% ± 3.54% when LSD1 was silenced by shRNA-LSD1-927 (Fig. 2C), indicating that achieving highly efficient differentiation to IPCs from hiPSCs is feasible. Moreover, the quantitative PCR-based gene expression profiling demonstrated that IPC differentiation induced by our approach closely parallels the key gene expression pattern of pancreas development in vivo (Fig. 2a). IPCs derived from hiPSCs with LSD1 knockdown by shRNA-LSD1-927 co-expressed mature pancreatic β cells markers insulin, MAFA, and glucokinase [27]. In in vitro glucose stimulation tests, we found that differentiated IPCs secreted less insulin or C-peptide, which was only 1/6–1/8 of that by adult human islets, suggesting that the IPCs that we obtained were not very mature to produce enough insulin (Fig. 2d). Recently, Kroon et al. transplanted human ES cell-derived pancreatic endoderm into immunodeficient mice and observed glucose-responsive insulin-secreting cells, suggesting that the in vitro-derived pancreatic precursors could mature in vivo [28]. Notably, these IPCs are generally not robust in glucose-stimulated insulin secretion, as shown by previously reported work. We observed that the morphology of these cells gradually collapsed (data not shown), the reason for which is unknown. We presume that this result was associated with oxidative stress based on the sensitivity of islet cells to oxygen free radicals [29]. In our studies, we transplanted differentiated IPCs-shRNA-LSD1-927 into immunodeficient SCID-Beige diabetic mice under the left kidney capsule and observed a drop in the blood sugar level. These results showed that IPCs were functional in vivo and could reverse hyperglycemia in diabetic mice. This newly established strategy suggests an efficient method to utilize patient-specific IPCs for the treatment of diabetes.

When LSD1 was silenced by shRNA-LSD1-927, hiPSCs could be efficiently induced to directionally differentiate into IPCs with increased efficiency and maturity. Therefore, we sought to determine a mechanism that could explain this shift. CoREST and HDAC1 are known to be found within LSD1 protein complexes in hiPSCs by Immunoprecipitation-Western blot (IP-WB) assay [30]. In Chip-qPCR, LSD1, CoREST, and HDAC1 were identified in almost the same promoter region of the developmental genes SOX17 and FOXA2 at approximately 100–2000 bp upstream of the TSS. This result indicates that LSD1 binds directly to the promoters of SOX17 and FOXA2 and regulates their expression. CoREST and HDAC1 are involved in the regulation process, which also supports the existence of the LSD1/CoREST/HDAC1 complex. When LSD1 was silenced by shRNA, CoREST expression in the promoter region of SOX17 and FOXA2 was unchanged, but HDAC1 expression was downregulated significantly. These results suggest that CoREST is involved in the demethylation process induced by LSD1 with no relationship to the LSD1 level, but that HDAC1 might be influenced directly or indirectly by the LSD1 level in an unknown way. When LSD1 was silenced, H3K4me2 level was significantly upregulated for the promoter regions of the endoderm genes SOX17 and FOXA2 (100–2000 bp upstream of the TSS) to activate the transcription of the genes directly. Furthermore, the upregulation of H3K4me3 levels in SOX17 and FOXA2 promoter regions would be expected to promote a significant increase in the gene expression levels.

Although OCT4 and SOX2 are not targets of LSD1, H3K4me3 levels in the promoter regions (1000–4000 bp upstream of TSS) were reduced dramatically, explaining the downregulation of the mRNA levels of these genes [31, 32]. The mechanism by which LSD1 regulates the expression of OCT4 and SOX2 remains unknown. LSD1-CoREST corepressor demethylates H3K4 in target gene promoters and then inhibits the occurrence of active histone modification H3K4 acetylation [33]. LSD1 inhibits the acetylation of H3K9 and further promotes the maintenance of transcriptional inactivation of target genes, which may be attributed to the existence of HDAC1 in the CoREST complex [34]. The acetylation of H3K9 in the promoter regions (100–2000 bp upstream of the TSS) of SOX17 and FOXA2 was upregulated significantly, strengthening the transcriptional activation of these genes. In summary, the upregulation of H3K4me2/me3 and H3K9act in the promoter regions of SOX17 and FOXA2 activates the early expression of these genes in combination and further promotes the highly efficient differentiation to IPCs [35].

## Conclusions

LSD1 activity regulates the balance between the proliferation and differentiation of hiPSCs. When LSD1 activity was 31.33%, hiPSCs skewed their development from proliferation to differentiation. hiPSCs with LSD1 silenced by shRNA-LSD1-927 differentiated mostly to endoderm after induction with higher efficiency and maturity and finally differentiated to mature pancreatic β-like cells. After transplantation to immunodeficient diabetic mice, these cells could secrete human insulin and reverse hyperglycemia. The underlying mechanism may be that LSD1 regulates histone methylation and acetylation in promoter regions of pluripotent genes and endoderm developmental genes, influences the ability to differentiate into endoderm, and finally directs highly efficient differentiation into IPCs.

## Supplementary information

**Additional file 1: Figure S1.** Effect of RNAi or LSD1 inhibitors on proliferation, cell cycle, and apoptosis in hiPSCs. A. hiPSC proliferation curves of the two groups were determined by CCK-8 assay. The proliferation index (OD values) are shown over a time-course (*P < 0.05; **P < 0.01, *n* = 3). B. Comparative analysis of cell apoptosis of two groups. The control group apoptosis rate was only 2.3% ± 0.43%. After treatment with LSD1 RNAi, the percentage of apoptosis cells was not significantly increased (*P* > 0.05, *n* = 3). C. After knocking down LSD1, we analyzed the cell cycle distribution of the control group (hiPSCs-scrambled-shRNA) and hiPSCs-shRNA-LSD1–927 group. D. When LSD1 activity was 31.3% (hiPSCs-shRNA-LSD1–927), cells arrested in the G0/G1 phase accounted for 46.3% ± 1.63%, which was higher than those of the hiPSCs-scrambled-shRNA group (*P* < 0.05, *n* = 3).

**Additional file 2: Figure S2.** Morphology of hiPSCs and expression levels of marker genes. A. hiPSC morphology under microscopy: morphology of clones from normal hiPSCs and hiPSCs-shRNA-LSD1–927 group (magnification: 100×). B. Real Time-PCR to detect gene levels after LSD1 inhibition with shRNAs (scrambled-shRNA, shRNA-LSD1–2495, shRNA-LSD1–863, shRNA-LSD1–927, and shRNA-LSD1–1086). OCT4, SOX2, and NANOG represent pluripotency genes, whereas SOX17, FOXA2, BMP2, and TUBB3 represent differentiation genes (*, *P <* 0.05; **, *P <* 0.01, *n* = 3).

**Additional file 3: Figure S3.** Flow cytometry analysis of differentiated hiPSCs (UMC1, UMC6). Flow cytometry analysis of differentiated hiPSCs revealed that after stage 1. A. 97.6% ± 2.3% of the UMC1 cells with shRNA-LSD1–927 were SOX17+ and CXCR4+ double-positive, and 88.6% ± 1.42% of the control cells (UMC1-scrambled-shRNA) were double-positive. A significant difference was observed between the two groups (shRNA-LSD1–927 and scrambled-shRNA) of UMC1 (*, *P* < 0.05, *n* = 3). B. 98.81% ± 1.54% of the UMC6 cells with shRNA-LSD1–927 were SOX17+ and CXCR4+ double-positive, and 87.5% ± 3.23% of the control cells (UMC1-scrambled-shRNA) were double-positive. A significant difference was observed between the two groups (shRNA-LSD1–927 and scrambled-shRNA) of UMC6 (*, *P* < 0.05, *n* = 3).

## Data Availability

The data supporting our findings can be found on online (http://yqq.szhospital.work/wap/download.php?channel_id=22197952&username=szrmyy). The download password is 123456.

## Abbreviations

hiPSCs: Human induced pluripotent stem cells
LSD1: Lysine-specific demethylase 1
IPCs: Insulin-producing cells
ESCs: Embryonic stem cells
DTZ: Dithizone
IP-WB: Immunoprecipitation-Western blot
TSS: Transcription start sites
