# Supplementary figures and images for: Inhibition of LSD1 promotes the differentiation of human induced pluripotent stem cells into insulin-producing cells
Source: Stem Cell Res Ther. 2020 May 19;11:185. doi: 10.1186/s13287-020-01694-8 (PMC7238565; doi:10.1186/s13287-020-01694-8)

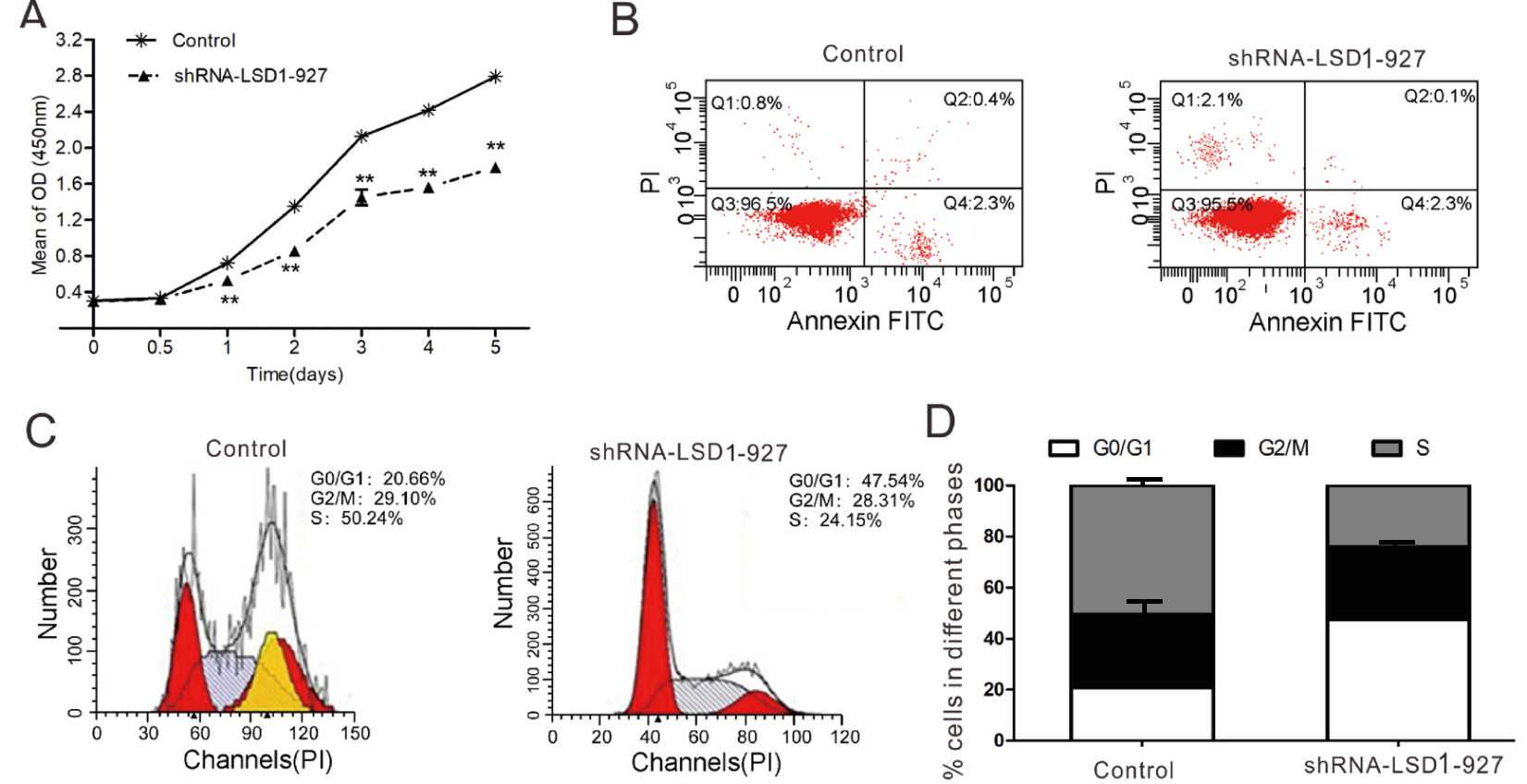

Supplement: Supplementary file 1 — Additional file 1: Figure S1. Effect of RNAi or LSD1 inhibitors on proliferation, cell cycle, and apoptosis in hiPSCs. A. hiPSC proliferation curves of the two groups were determined by CCK-8 assay. The proliferation index (OD values) are shown over a time-course (*P < 0.05; **P < 0.01, n = 3). B. Comparative analysis of cell apoptosis of two groups. The control group apoptosis rate was only 2.3% ± 0.43%. After treatment with LSD1 RNAi, the percentage of apoptosis cells was not significantly increased (P > 0.05, n = 3). C. After knocking down LSD1, we analyzed the cell cycle distribution of the control group (hiPSCs-scrambled-shRNA) and hiPSCs-shRNA-LSD1–927 group. D. When LSD1 activity was 31.3% (hiPSCs-shRNA-LSD1–927), cells arrested in the G0/G1 phase accounted for 46.3% ± 1.63%, which was higher than those of the hiPSCs-scrambled-shRNA group (P < 0.05, n = 3). [file 13287_2020_1694_MOESM1_ESM.tif]

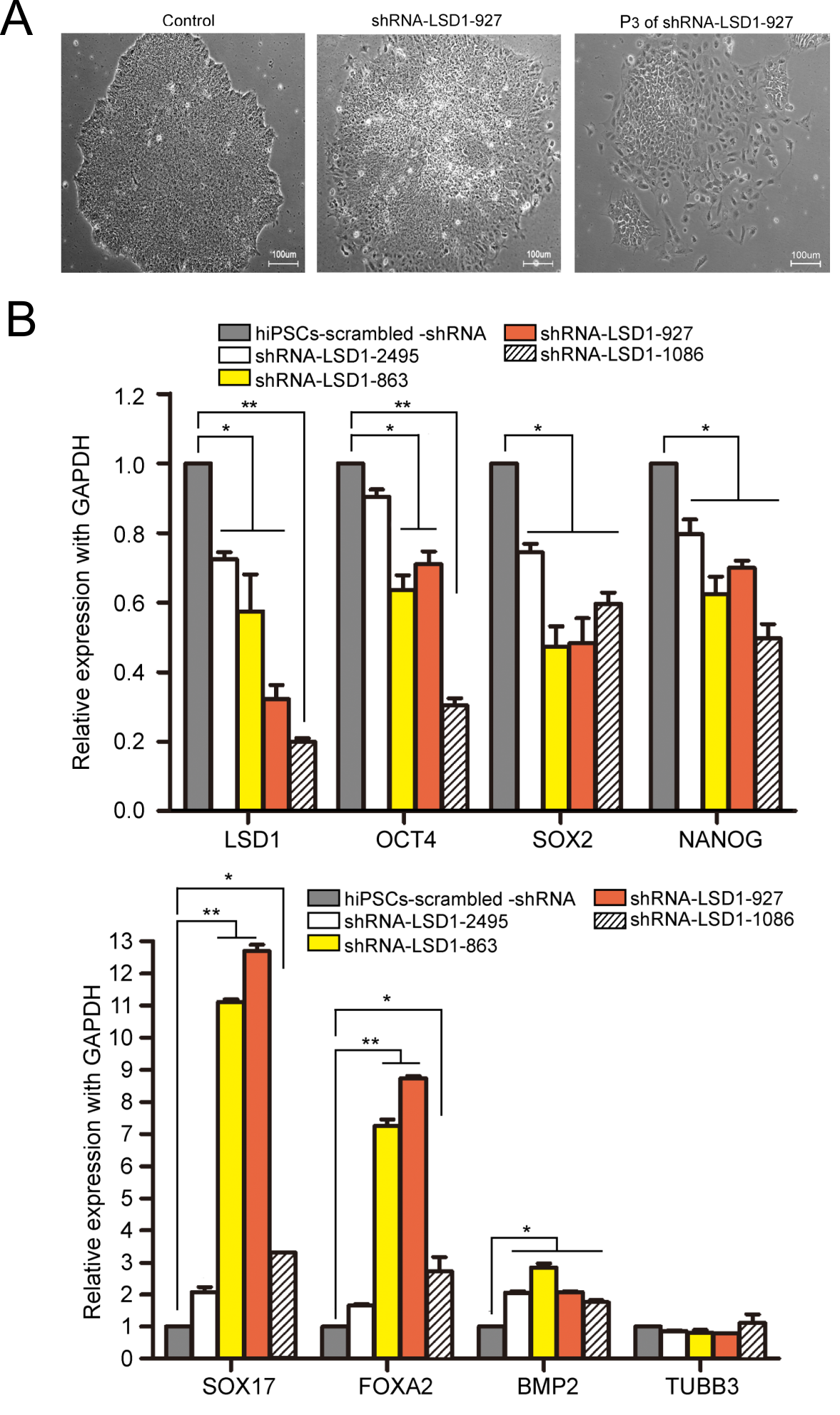

Supplement: Supplementary file 2 — Additional file 2: Figure S2. Morphology of hiPSCs and expression levels of marker genes. A. hiPSC morphology under microscopy: morphology of clones from normal hiPSCs and hiPSCs-shRNA-LSD1–927 group (magnification: 100×). B. Real Time-PCR to detect gene levels after LSD1 inhibition with shRNAs (scrambled-shRNA, shRNA-LSD1–2495, shRNA-LSD1–863, shRNA-LSD1–927, and shRNA-LSD1–1086). OCT4, SOX2, and NANOG represent pluripotency genes, whereas SOX17, FOXA2, BMP2, and TUBB3 represent differentiation genes (*, P < 0.05; **, P < 0.01, n = 3). [file 13287_2020_1694_MOESM2_ESM.tif]

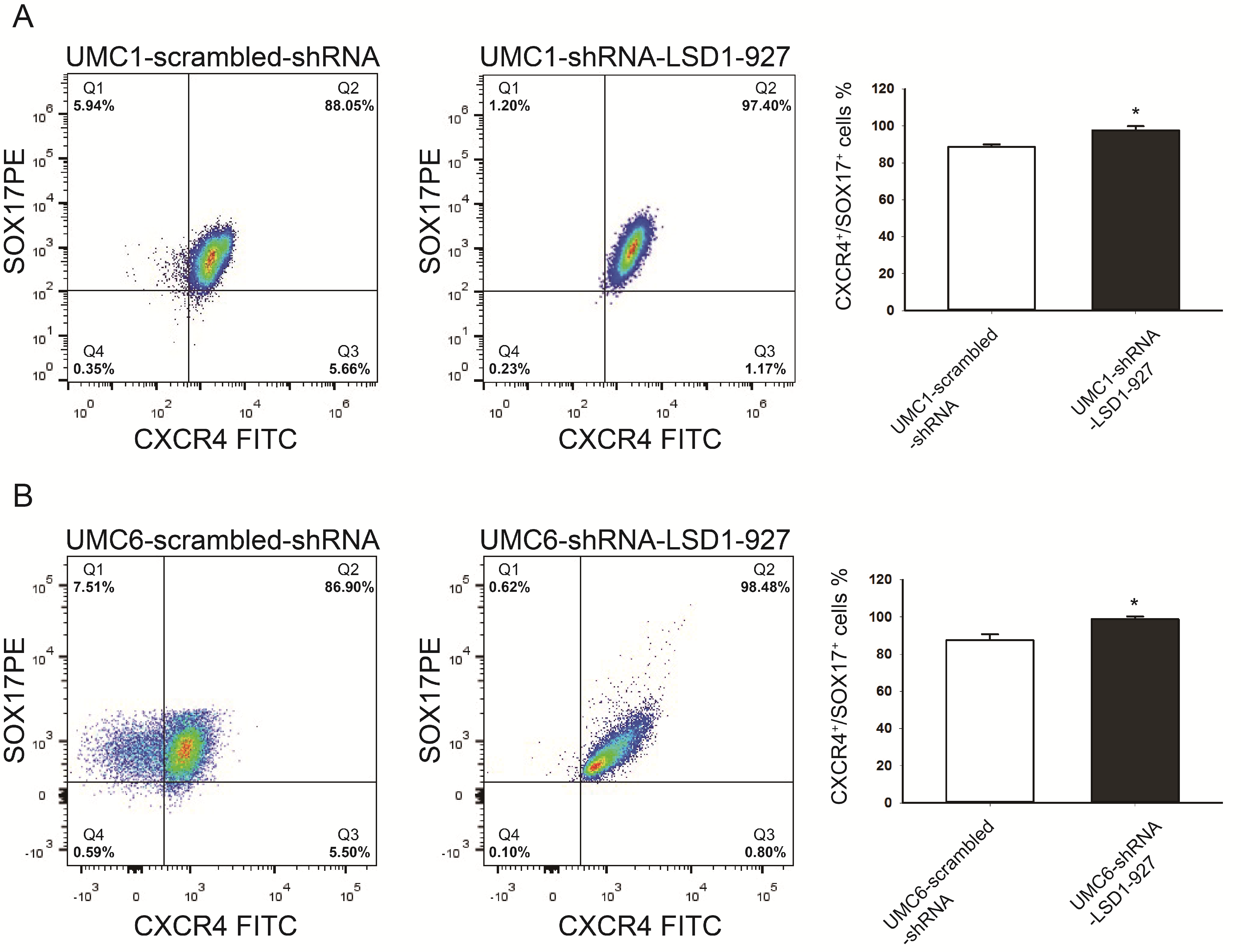

Supplement: Supplementary file 3 — Additional file 3: Figure S3. Flow cytometry analysis of differentiated hiPSCs (UMC1, UMC6). Flow cytometry analysis of differentiated hiPSCs revealed that after stage 1. A. 97.6% ± 2.3% of the UMC1 cells with shRNA-LSD1–927 were SOX17+ and CXCR4+ double-positive, and 88.6% ± 1.42% of the control cells (UMC1-scrambled-shRNA) were double-positive. A significant difference was observed between the two groups (shRNA-LSD1–927 and scrambled-shRNA) of UMC1 (*, P < 0.05, n = 3). B. 98.81% ± 1.54% of the UMC6 cells with shRNA-LSD1–927 were SOX17+ and CXCR4+ double-positive, and 87.5% ± 3.23% of the control cells (UMC1-scrambled-shRNA) were double-positive. A significant difference was observed between the two groups (shRNA-LSD1–927 and scrambled-shRNA) of UMC6 (*, P < 0.05, n = 3). [file 13287_2020_1694_MOESM3_ESM.tif]
